# A high oleic sunflower oil fatty acid esters of plant sterols mixed with dietary diacylglycerol reduces plasma insulin and body fat accumulation in *Psammomys obesus*

**DOI:** 10.1186/1476-511X-8-42

**Published:** 2009-10-12

**Authors:** Ehud Ziv, Natan Patlas, Rony Kalman, Dori Pelled, Yael Herzog, Tali Dror, Tzafra Cohen

**Affiliations:** 1 Diabetes Research Unit, Hadassah Medical Center and The Hebrew University, Jerusalem 91120, Israel; 2 Animal facility, Hebrew University, Jerusalem, Israel; 3 R&D Department, Enzymotec LTD, Migdal-HaEmeq, Israel; 4 Current address: Medical Department, NiTi Surgical solutions, Netanya, Israel

## Abstract

**Background:**

Metabolic syndrome is associated with subsequent development of cardiovascular diseases and type 2 diabetes. It is characterized by reduced response to insulin, central obesity, and dyslipidemia. Intake of plant sterols (PS) has been shown to confer a healthier lipid profile and ameliorate cardiovascular disease risk factors in experimental animals and humans. In this study we used an animal model of type 2 diabetes to assess the effects of a preparation of PS esterified to high oleic sunflower oil fatty acids mixed with dietary diacylglycerol (PS-HOSO) on diabetic related metabolic parameters. *Psammomys obesus *(*P. obesus*) were fed high energy (HE) diet supplemented by either PS-HOSO or control oil. Following 4.5 weeks of intervention, animals were divided into fasting and non-fasting modes prior to outcome measurements. Glucose and insulin levels as well as blood lipid profile, body weight, and fat accumulation were evaluated in fasting and non-fasting modes.

**Results:**

*P. obesus *fed with a HE diet displayed a characteristic heterogeneity in their blood glucose and insulin levels with a subset group displaying type 2 diabetes symptoms. PS-HOSO treatment significantly reduced total cholesterol (24%, *P *< 0.001) and non-HDL cholesterol (34%, *P *< 0.01) compared to the control diet. Among fasting animals, body weight at end point and epididymal fat-to-liver weight ratio were significantly (*P *< 0.05 each) reduced (7% and 16%, respectively) compared to controls. Interestingly, fasting blood glucose levels were similar between groups, whereas plasma insulin level at end point was 44% lower in the PS-HOSO group compared to control group (*P *< 0.0001)

**Conclusion:**

PS-HOSO supplementation to diabetes-prone gerbils counteracts the increase in body weight and epididymal fat accumulation, and also results in a drop in circulating insulin levels. These effects are pointing out that PS-HOSO may serve as a functional ingredient for metabolic syndrome or diabetic sufferers, which not only influences body weight, but also prevents or reverses insulin resistance and hyperlipidemia.

## Background

Metabolic syndrome represents a cluster of metabolic disturbances that attained almost epidemic proportions worldwide and is associated with subsequent development of type 2 diabetes mellitus and cardiovascular disease [1,2]. It is characterized by central obesity [3,4], hypertension, hyperglycemia and reduced response to insulin (insulin resistance). In addition, metabolic syndrome sufferers display various degrees of dyslipidemia. The main lipid metabolism abnormalities seen in these subjects are increased plasma triglycerides (TG) and reduced high-density lipoprotein cholesterol (HDL-c) levels, along with a predominance of small low-density lipoprotein (LDL) particles [5]. It has therefore been proposed that alteration of the fat metabolism is a critical factor that may precipitate the etiology of the disease, at least in some cases. Favourable modifications of the lipid profile in sufferers of metabolic syndrome or type-2 diabetes, may be achieved by pharmaceutical means [6,7]. Recently, dietary supplements and functional foods have been increasingly used as lipid-lowering agents [8,9]. Intake of the n-9 monounsaturated fatty acid, oleic acid, was shown to be inversely correlated with ischemic heart disease presumably due to hypolipidemic effects [10]. This notion was further confirmed in several clinical intervention trials [11-13]. Plant sterols (PS) were also reported to have significant cholesterol-lowering properties [14]. Accordingly, PS Intake decreased circulating cholesterol concentrations via the suppression of intestinal absorption due to the higher affinity of PS to micelles [15,16]. Recent works have shown that PS incorporated into food matrices such as margarines and spreads or in the form of dietary supplements are bioactive for long term treatment periods [17,18].

Israeli sand rat *Psammomys obesus*, (*P. obsesus*), a desert gerbil species has been extensively used as a genetically heterogeneous model for type 2 diabetes. *P. obesus *closely resembles some human populations in which a subset of the population goes on to develop diabetes when fed a high energy diet [19,20]. The course of the disease development in these animals can be divided into four phases: Phase A, normoinsulinemia and normoglycemia; phase B, hyperinsulinemia and normoglycemia; phase C, overt diabetes, characterized by hyperinsulinemia and hyperglycemia; and phase D, hypoinsulinemia and severe hyperglycemia as a result of β-cell degranulation, and markedly reduced pancreatic insulin content [21]. As in humans, obesity and increase in body fat underlie the development of type 2 diabetes.

We have previously shown the efficacy of preparations of PS esterified to high oleic oil fatty acids in reducing total cholesterol (TC) levels in experimental animals [22] and humans [23]. In addition, the preparations were shown to reduce plasma lipid peroxidation.

In the present study, we investigated the effect of PS esterified to high oleic sunflower oil (HOSO) fatty acids mixed with dietary diacylglycerol (DAG) in HOSO matrix (PS-HOSO) or soybean oil on the development of metabolic syndrome and diabetic symptoms in *P. obesus*.

## Methods

### Animals and diets

Sixty male *Psammomys obesu*s gerbils were obtained from Harlan Laboratories Ltd (Jerusalem, Israel). After weaning, the animals were maintained on a low-energy diet containing 2.38 kcal/g (Koffolk, Petach Tikva, Israel) by Harlan Laboratories Ltd. Our experiment started when the animals were 2.0 to 3.5 months old. The animals were then randomly assigned to two different high-energy diets for 4.5 weeks:

1. Control HE (high energy) diet: 938.5 g/Kg version of 2018SC+F Harlan Tekled Ltd, USA, supplemented with soybean oil to a final fat concentration of 6%.

2. PS-HOSO diet (custom-made, Harlan Tekled Ltd, USA): 939 g/Kg version of 2018SC+F, Harlan Tekled Ltd, USA, supplemented with PS-HOSO to a final fat concentration of 6%. PS-HOSO constituted 4% w/w of total diet and comprised 20% plant sterols esterified with high oleic sunflower oil, and 15% canola oil-based diacylglycerol (DAG).

This diet was made from a slightly concentrated version of 2018CS+F with 2% fat source basal mix. Standard milling procedure was used to incorporate treatment oil into standard gerbil chow.

The animals were housed in polypropylene cages at a constant temperature of 22 to 23°C in a humidity-controlled animal facility with a 12-h light/dark cycle. Free access to water and food was provided. The animals were monitored for body weight and tail blood glucose concentrations, from the beginning of the intervention and throughout (every 2-5 days), for 4.5 weeks. At endpoint, half of each group of gerbils was deprived of food overnight (16 h), and then all animals were lightly anaesthetized with ketamine (Ketalar; Parke-Davis & Co., United Kingdom) and exsanguinated by cardiac puncture. Blood samples were collected for biochemical analyses. The liver and epididymal fat were harvested and weighted. All experimental procedures performed in the study were authorized by the Institutional Animal Care and Use Committee.

### Biochemical measures

Blood glucose concentration was determined by the enzymatic glucose analyzer, Glucometer Elite (Bayer, Elkhart, IN) on blood samples taken from the tail vein. Plasma samples that were obtained from EDTA-treated blood samples by separation at 12,000 × g for 15 min, were stored until lipid analysis, at -80°C. Then, an aliquot was analyzed for total cholesterol, TG and HDL-cholesterol concentrations by colorimetric methods (Boehringer Mannheim, Mannheim, Germany). Insulin levels in plasma were assessed by radioimmunoassay using a human primary antibody (Phadesph; Kabi Pharmacia Diagnostics, Uppsala, Sweden) according to the manufacturer's instructions.

### Lipid extraction from liver samples

The liver samples were weighed and homogenized with saline at a ratio of 1:5 (w:v) in plastic tubes on ice. Lipids were extracted from an aliquot of the liver homogenate according to the procedure of Folch *et al*. [24]. The total amount of fat was calculated according to Leikin-Frenkel *et al*. [25].

### Data Analysis

Data was expressed as means ± SD and statistical significance was taken as *P *< 0.05. Data was analyzed by one-way analysis of variance (ANOVA) model for continuous variables to assess the differences among the experimental groups. Fisher exact test was used for comparison of nonparametric variables, namely, gerbil survival, and the number of gerbils in phase C, D, or presenting normoglycemia. The assumption of normal distribution and homogeneity of variance was tested for all outcome measures. Accordingly, the differences between groups were evaluated by two-tailed Student's *t*-test or Wilcoxon's signed-rank test. Spearman's rank-order correlation coefficient was used to evaluate the associations between changes in insulin and in blood glucose levels at the intervention endpoint. The statistical analyses were carried out by using SPSS statistical software (SSPS Inc, Chicago, IL) version 13.0.

## Results

### Animals

By the end of the 4.5 weeks intervention, 3 gerbils from the control group had normal blood glucose levels (below 140 mg/dl), 22 presented typical phase C profile, one gerbil presented typical phase D symptoms (triglyceride levels above 500 mg/dl) and 4 gerbils had died from unrelated causes. Similarly, within the PS-HOSO diet group, 6 animals had normal blood glucose levels, 19 demonstrated phase C characteristics, 4 gerbils presented typical phase D symptoms, and one gerbil died. There were no statistically significant differences between the two groups in the numbers of animal in phase C, phase D, animal with normal glucose levels, or dead animals. The baseline body weights and blood glucose levels of the gerbils are shown in Table 1. No significant differences among the various groups were evident.

**Table 1:** Baseline characteristics of tested gerbils^1^.

	**All tested gerbils**						**Gerbils in phase C**					
	**Control**			**PS-HOSO**			**Control**			**PS-HOSO**		

	**Total**	**Non-fasting**	**Fasting**	**Total**	**Non-fasting**	**Fasting**	**Total**	**Non-fasting**	**Fasting**	**Total**	**Non-fasting**	**Fasting**

**n**	**30**	**16**	**14**	**30**	**14**	**16**	**22**	**10**	**12**	**19**	**8**	**11**

Body weight (gr)	162.40 ± 22.7	156.94 ± 24.5	168.64 ± 19.5	163.93 ± 26.2	169.43 ± 32.4	159.13 ± 19.2	167.14 ± 20.2	163.6 ± 25.7	170.08 ± 14.6	159.21 ± 27.5	160.50 ± 35.9	158.27 ± 21.3

Glucose (mg/dL)	73.97 ± 8.0	72.63 ± 6.9	75.50 ± 9.2	72.40 ± 10.1	76.93 ± 11.0	*68.44 ± 7.5	73.77 ± 7.2	72.30 ± 7.6	75.00 ± 7.0	72.05 ± 9.6	75.75 ± 11.5	69.36 ± 7.3

*Psammomys obesus *were weighted and tested for blood glucose levels at the beginning of the experiment. Following 4.5 weeks of experimental diet feeding, half of each animal group were deprived of food for 16 h. Statistical significance between baseline values of control and PS-HOSO treatments was calculated for the gerbils that started the feeding period (all gerbils, total), for the subgroup that was later assigned to fast (all gerbils, fasting) and the subgroup that was not assigned to fast (all gerbils, non-fasting). Statistical significance was also calculated for gerbils that were in phase C at endpoint (phase C, total), for phase C gerbils that were not assigned to fast (phase C, non-fasting) and for phase C gerbils that were assigned to fast (phase C, fasting).

^1^Values are means ± S.D

* *P *< 0.05.

PS-HOSO: plant sterol esterified with high oleic sunflower oil.

### Body weight, liver weight and fat accumulation of phase C gerbils

PS-HOSO effect on body weight, liver weight and fat accumulation is shown in Table 2. Animals fed PS-HOSO and assigned to the fasting mode reduced (*P *= 0.045) 6.7% in their body weight as compared to the control diet fed animals. The relative amount of white adipose tissue, as determined by the ratio (w/w) of epididymal fat to liver decreased (*P *= 0.044) by 16% compared to the corresponding control group. None of the above mentioned effects were observed in the non-fasting group. Liver weight at the end of the study was 11% smaller (*P *= 0.039) among non-fasting animals in the control group, as compared to non-fasting animals in the PS-HOSO group. This difference was abolished in the fasting groups. Analysis of liver fat weight to liver weight ratio didn't reveal any significant difference between treatment groups among non-fasting animals, indicating that the liver weight difference didn't result from liver fat accumulation. Interestingly, among fasting animals, this ratio was 27% lower (*P *= 0.04) in the PS-HOSO group in comparison with that of the control group.

**Table 2:** PS-HOSO effect on body weight, liver weight and fat accumulation of phase C gerbils^1^.

	**Control**			**PS-HOSO**		
	**Total**	**Non-fasting**	**Fasting**	**Total**	**Non-fasting**	**Fasting**

Bodyweight at endpoint (gr)	219.05 ± 16.2	214.60 ± 15.8	222.75 ± 16.2	211.58 ± 19.8	216.63 ± 23.0	* 207.91 ± 17.3

Liver weight (gr)	8.23 ± 0.77	8.36 ± 0.80	8.12 ± 0.77	8.68 ± 1.19	* 9.40 ± 1.17	8.15 ± 0.93

Liver fat weight to liver weight ratio	0.11 ± 0.03	0.10 ± 0.02	0.11 ± 0.04	* 0.08 ± 0.01	0.08 ± 0.08	* 0.08 ± 0.01

Epididymal fat weight (gr)	6.35 ± 0.94	6.25 ± 0.96	6.43 ± 0.95	5.88 ± 1.73	6.54 ± 2.30	* 5.41 ± 1.06

Epididymal fat to liver weight ratio	0.78 ± 0.14	0.75 ± 0.12	0.80 ± 0.15	0.68 ± 0.18	0.69 ± 0.25	* 0.67 ± 0.14

*Psammomys obesus *were fed with Control or PS-HOSO as described in the method section and were monitored for body weight. Following 4.5 weeks of experimental diet feeding, half of each animal group was deprived of food for 16 h and then all animals were weighted and exsanguinated. The liver and epididymal fat were harvested and weighted. Liver fat of 15 PS-HOSO treated animals (9 and 6 from the fasting and non-fasting groups, respectively) and 16 control treated animals (7 and 9 from the fasting and non-fasting groups, respectively) was extracted and weighted.

Statistical significance between endpoint values of control and PS-HOSO treatments were calculated for all of phase C gerbils (total), and for fasting and non-fasting phase C gerbils.

^1^Values are means ± S.D

*P < 0.05.

PS-HOSO: plant sterol esterified with high oleic sunflower oil.

### Biochemical measures of Phase C gerbils

**Figure 1:**
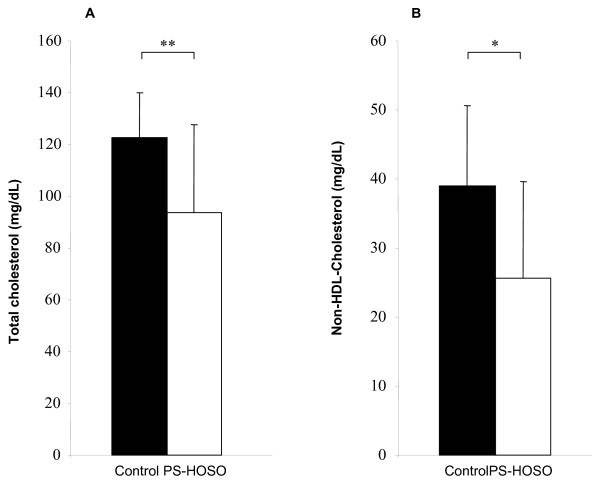
**Effect of PS-HOSO on plasma lipid levels of phase C gerbils**. *Psammomys obesus *were fed with control (closed bars) or PS-HOSO (open bars) as described in the method section. Following 4.5 weeks of experimental diet feeding, total cholestrol (A) and non-HDL cholesterol (B) were measured. Results are means ± SD. Statistical significance between endpoint values of control and PS-HOSO treatments are * P-Value < 0.01, ** P-Value < 0.001. PS-HOSO: plant sterol esterified with high oleic sunflower oil.

Treatment with PS-HOSO significantly reduced the plasma levels of TC and non-HDL cholesterol by 24% (*P *= 0.001) and 34% (*P *= 0.002), respectively, compared to the control diet (Figure 1). When the fasting and non-fasting groups of phase C gerbils were analyzed separately, total cholesterol was reduced (*P *= 0.002) by 28% in the PS-HOSO treated non-fasting animals, and tended to decrease (*P *= 0.16) by 20.5% in the fasting group, as compared to the control-fed animals (data not shown). Non-HDL cholesterol concentrations tended to decrease (*P *= 0.064) by 37% in the PS-HOSO treated non-fasting animals, and decreased (*P *= 0.049) by 31% in the fasting animals, as compared to the control groups (data not shown).

No significant differences in blood glucose levels between the PS-HOSO-fed animals and the control diet-fed animals were detected at the end of the experimental diets intake (Table 3). The glucose levels among all of phase C gerbils, prior to fasting assignment, were elevated (280-340 mg/dl) as indicated by the three last glucose measurements (data not shown). The plasma levels of insulin dropped (*P *< 0.0001) by 39% at the endpoint of the experiment in the phase C PS-HOSO group, compared to the control (Table 3). Comparable findings were shown when the fasting and non-fasting groups were analyzed separately in the fasting (44%; *P *< 0.0001) and non-fasting (33%; *P *= 0.014) groups.

**Table 3:** PS-HOSO effect on glucose and insulin levels of phase C gerbils^1^.

	**Control**			**PS-HOSO**		
	**Total**	**Non-fasting**	**Fasting**	**Total**	**Non-fasting**	**Fasting**

Glucose at endpoint (mg/dL)	169.41 ± 106.7	271.70 ± 62.2	84.17 ± 33.4	180.16 ± 136.6	296.38 ± 143.0	95.64 ± 24.6

Insulin at endpoint (μIu/L)	211.36 ± 61.0	228.60 ± 67.1	197.00 ± 54.0	*** 128.37 ± 45.7	* 153.63 ± 40.3	*** 110.00 ± 41.6

*Psammomys obesus *were fed with control or PS-HOSO as described in the method section and were monitored for blood glucose levels. Following 4.5 weeks of experimental diet feeding, half of each animal group was deprived of food for 16 h and then all animals were tested for blood glucose and insulin levels. Statistical significance between endpoint values of control and PS-HOSO treatments were calculated for all of phase C gerbils (total), and for fasting and non-fasting phase C gerbils.

^1^Values are means ± S.D

* *P *< 0.05., ** *P *< 0.0001.

PS-HOSO: plant sterol esterified with high oleic sunflower oil.

**Figure 2:**
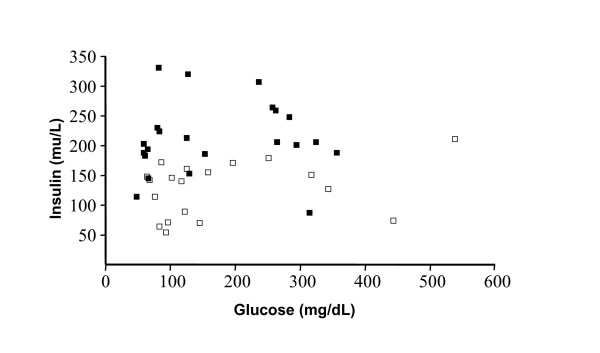
**Effect of PS-HOSO on plasma insulin levels versus blood glucose levels of phase C gerbils**. *Psammomys obesus *were fed with control (closed squares) or PS-HOSO (open squares) as described in the method section. Following 4.5 weeks of experimental diet feeding, the correlation between plasma insulin and blood glucose levels was tested. There was no correlation between plasma insulin and blood glucose levels among PS-HOSO fed animals (*r *= 0.318; *n *= 19; *P *= 0.185), nor among control fed animals (*r *= 0.17; *n *= 22; *P *= 0.448). PS-HOSO: plant sterol esterified with high oleic sunflower oil.

In order to test the possibility that the insulin level reduction observed in the PS-HOSO gerbils was caused by β-cell degranulation and deterioration toward phase D, we plotted plasma insulin vs. blood glucose for each separate animal (Figure 2). There was no correlation between the insulin levels and the glucose levels in PS-HOSO animals, regardless of the feeding regime, indicating that the insulin reduction is probably not caused by β-cell degranulation. Another indication for the animals not being in phase D is the fact that they were screened for blood TG levels of below 500 mg/dl.

The effect of PS-HOSO feeding on the insulin levels is strikingly evident from Figure 2 as the insulin measurement values of the PS-HOSO-fed animals cluster in the bottom end of the scale, compared to those of the control diet-fed animals.

## Discussion

The aim of this study was to test the effect of PS-HOSO, a novel preparation of plant sterols esterified to high oleic sunflower oil fatty acids mixed with 1,3-DAG, in a matrix of high oleic sunflower oil, on the development of metabolic syndrome and diabetic symptoms in *Psammomys obesus*. PS have been intensely studied in recent years and have shown to lower TC and LDL-C. These effects are obtained mainly by reducing the intestinal absorption of dietary and biliary cholesterol [26,9]. Current hypotheses ascribe a vital role to the dysfunction of lipid metabolism in the development of metabolic syndrome and type 2 diabetes [27,28]. Thus, we chose to test the effect of PS-HOSO preparation in the gerbil *Psammomys obesus *which serves as a model of nutritionally induced type 2 diabetes. The progression of metabolic syndrome, and the subsequent diabetes mellitus, in these animals, resembles in many aspects the development of metabolic syndrome and diabetes in susceptible humans [19,20].

In this study, PS-HOSO treatment significantly reduced the TC and non-HDL cholesterol among phase C gerbils, compared to control diet treatment. These results are in agreement with an animal study performed on Apolipoprotein E-deficient mice [22] which demonstrated that administration of PS esters of canola oil fatty acids in a canola oil matrix strongly tended to lower TC levels. Similarly, a human study performed on hypercholesterolemic, mildly overweight individuals [23] showed that administration of PS esterified to olive oil in an olive oil based matrix, significantly reduced LDL levels. These results indicate that PS maintains their cholesterol lowering properties when esterified to high oleic sunflower oil fatty acids.

Interestingly, treatment with PS-HOSO significantly reduced body weight and the amount of epididymal fat among fasting phase C gerbils in comparison with the control group. The reason for this reduction is not clear, however, 1,3-DAG, a component of PS-HOSO, has been previously reported to reduce the extent of postprandial TG increase [29] and to reduce body fat in obese individuals [30]. It is assumed that 1,3-DAG-rich diet is less readily reesterified into chylomicron-TG moiety, thereby reducing the transport of dietary neutral lipids to the blood circulation and their deposit in adipose tissue [31]. Instead, 1,3-DAG may be devoted to substantially enhance β-oxidation, resulting in a decrease in abdominal body fat and weight [32-34]. However, these results were obtained following administration of higher doses of 1,3-DAG than the present study dosage and thus, the mechanism of by which PS-HOSO reduce fat accumulation remains to be elucidated. It should be noted that liver weight among phase C, non-fasting gerbils in the PS-HOSO group was greater than that of the non-fasting gerbils in the control group. However, the possibility that PS-HOSO treated animals developed a fatty liver was excluded by measuring the amount of liver total fat. There was no significant difference between the non-fasting groups in the ratio of liver fat weight to liver weight. The difference in liver weight at non-fasting state may be explained by modifications in postprandial hepatic glycogen metabolism. Human studies have shown that after meal, glycogen accumulation in type 2 diabetic patients rise to a lesser extent in comparison with non-diabetic subjects [35,36]. Thus, greater liver weight may even indicate an ameliorating effect of PS-HOSO on the type 2 diabetic metabolic defects, influencing hepatic glycogen storage. In addition, glycogen metabolic differences, as the cause for liver weight difference between the groups, may be further supported by the fact that, among fasting groups, the described difference in liver weight between PS-HOSO and control groups was abolished. In fact, in this group, the ratio of liver fat weight to liver weight was smaller among PS-HOSO treated animals in comparison with control treated animals, indicating the possible role of PS-HOSO in reducing liver fat accumulation.

It is well established today that adipose tissue does not function solely as an energy storage compartment, but it is also endowed with diverse regulatory roles thanks to a complex system of endocrine regulation that affects energy intake and distribution [37]. Increases in body fat mass are accompanied by elevation of free fatty acids, leptin and insulin [38,39,33]. In the present study, PS-HOSO did not only affect fat accumulation and storage, but it also caused a dramatic reduction in circulating insulin levels. Several studies that analyzed the effects of plant sterols in type 2 diabetic subjects did not report insulin levels [40-42], and, to the best of our knowledge, this is the first investigation that deals with the impact of phytosterol on insulin concentrations. It is possible that the insulin reduction can be attributed to the presence of 1,3-DAG since previous studies emphasized its relationship with serum insulin reduction both in animal models [33] and subjects with type 2 diabetes mellitus [43]. Although the amount of DAG in PS-HOSO is relatively low, compared to the amount used in the above-mentioned studies, it is still possible that some synergistic effect between DAG and the plant sterols takes place.

In addition, it was recently shown that oleate might have a role in protecting against palmitate-induced inflammation and insulin resistance in skeletal muscle cells [44]. It is thus possible that the oleic acid in the PS-HOSO preparation contributes as well to the significant insulin reduction recorded with PS-HOSO.

In contrast to the reduction in insulin levels, fasting blood glucose levels (glucose at endpoint) remained unchanged in the PS-HOSO treated gerbils compared to the control diet fed gerbils. This confirms the results from a published phytosterol study in subjects with type 2 diabetes in which no change was detected in blood glucose levels, despite lipid profile improvement [41]. Hypoinsulinemia in combination with hyperglycemia are characteristics of phase D gerbils, however, as shown in Figure 2 there was no correlation between blood glucose levels and plasma insulin levels among PS-HOSO fed gerbils. In addition, the animals were clearly not in phase D, since their triglyceride levels were not elevated.

## Conclusion

Hyperinsulinemia is a hallmark of metabolic syndrome and type 2 diabetes and it is necessary to keep it under control. We show here that PS-HOSO, as a dietary supplement, can help achieve this aim together with other desirable therapeutic goals such as weight loss, fat reduction and the lowering of blood cholesterol levels. These data warrant further evaluation of PS-HOSO as a functional ingredient for pre-diabetic populations.

## Competing interests

DP, TC are Directors of Clinical Studies and YH, TD are Project Managers at Enzymotec LTD. EZ, NP and RK have no competing interests. This study was funded by Enzymotec LTD, Israel.

## Authors' contributions

EZ designed the study. NP and RK carried out all aspects of the animal studies. DP participated in the design of the study, animal studies and performed statistical analysis. YH was responsible for drafting the manuscript and participated in data analyses. TD participated in drafting the manuscript, and data analyses. TC participated in the design of the study, participated in animal studies and data analyses. All authors participated in data interpretation. All authors have read and approved this manuscript.
